# MRI-based radiomics for preoperative T-staging of rectal cancer: a retrospective analysis

**DOI:** 10.1007/s00384-025-04969-9

**Published:** 2025-08-08

**Authors:** Vittorio Patanè, Umberto Atripaldi, Mario Sansone, Luca Marinelli, Sara Del Tufo, Gianluca Arrichiello, Davide Ciardiello, Francesco Selvaggi, Erika Martinelli, Alfonso Reginelli

**Affiliations:** 1 Department of Precision Medicine, University of Campania “Luigi Vanvitelli”, Piazza Luigi Miraglia 2, 80138 Naples, Italy; 2 Department of Electrical Engineering and Information Technology, University of Naples “Federico II”, 80125 Naples, Italy; 3 Oncology Unit, AORN “S. Anna e S. Sebastiano”, Caserta, Italy; 4 Division of Gastrointestinal Medical Oncology and Neuroendocrine Tumors, European Institute of Oncology (IEO), IRCCS, Milan, Italy; 5 Department of Advanced Medical and Surgical Sciences, Università Degli Studi Della Campania “Luigi Vanvitelli”, Naples, Italy

**Keywords:** Rectal cancer, MRI, Radiomics, Artificial Intelligence, Oncologic Imaging

## Abstract

**Puropose:**

Preoperative T-staging in rectal cancer is essential for treatment planning, yet conventional MRI shows limited accuracy (~ 60–78). Our study investigates whether radiomic analysis of high-resolution T2-weighted MRI can non-invasively improve staging accuracy through a retrospective evaluation in a real-world surgical cohort.

**Methods:**

This single-center retrospective study included 200 patients (January 2024–April 2025) with pathologically confirmed rectal cancer, all undergoing preoperative high-resolution T2-weighted MRI within one week prior to curative surgery and no neoadjuvant therapy. Manual segmentation was performed using ITK‑SNAP, followed by extraction of 107 radiomic features via PyRadiomics. Feature selection employed mRMR and LASSO logistic regression, culminating in a Rad-score predictive model. Statistical performance was evaluated using ROC curves (AUC), accuracy, sensitivity, specificity, and Delong’s test.

**Results:**

Among 200 patients, 95 were pathologically staged as T2 and 105 as T3–T4 (55 T3, 50 T4). After preprocessing, 26 radiomic features were retained; key features including ngtdm_contrast and ngtdm_coarseness showed AUC values > 0.70. The LASSO-based model achieved an AUC of 0.82 (95% CI: 0.75–0.89), with overall accuracy of 81%, sensitivity of 78%, and specificity of 84%.

**Conclusion:**

Radiomic analysis of standard preoperative T2-weighted MRI provides a reliable, non-invasive method to predict rectal cancer T-stage. This approach has the potential to enhance staging accuracy and inform personalized surgical planning. Prospective multicenter validation is required for broader clinical implementation.

## Introduction

Colorectal cancer (CRC) is among the most prevalent malignancies worldwide and remains a leading cause of cancer-related mortality [1–4]. Rectal cancer accounts for approximately one-third of CRC cases and requires a multidisciplinary approach to optimize outcomes [5–8]. Treatment strategies are determined based on local tumor extent, lymph node involvement, and the presence or absence of distant metastases [9, 10]. According to the National Comprehensive Cancer Network (NCCN) guidelines, patients with locally advanced rectal cancer (LARC)—defined as tumors staged clinically as T3, T4, or node-positive without distant spread—are typically managed with neoadjuvant chemoradiotherapy (nCRT), followed by total mesorectal excision (TME) and adjuvant chemotherapy [11–16].

Accurate local staging is essential to guide treatment decisions, particularly regarding the depth of tumor invasion (T stage), which determines whether patients may undergo immediate surgery or require preoperative therapy [17–20]. Magnetic resonance imaging (MRI) is the current gold standard for local staging, offering superior soft-tissue contrast and enabling assessment of tumor infiltration, mesorectal fascia (MRF), extramural vascular invasion (EMVI), sphincters, and the pelvic sidewall [21–25]. The multiplanar capabilities of MRI further support detailed tumor delineation and surgical planning [26–28].

Despite its advantages, conventional MRI exhibits variable diagnostic accuracy for T staging, ranging from 60 to 78%, depending on imaging quality, radiologist experience, and tumor morphology [29–31]. Purely morphological assessments may miss subtle heterogeneity in tumor behavior, prompting growing interest in quantitative imaging biomarkers to enhance staging precision [32–35].

Radiomics—a computational approach that extracts high-dimensional features from medical images—has emerged as a powerful tool to augment the diagnostic potential of standard imaging [36–40]. By quantifying tumor shape, texture, intensity, and spatial complexity, radiomic features offer a reproducible and objective insight into tumor biology, enabling their integration into predictive models for clinical decision-making [41–44].

In rectal cancer, radiomics has shown promise in multiple areas, including prediction of nCRT response, nodal staging, prognosis, and T-stage classification [41, 45–48]. However, most studies focus on post-treatment evaluation or rely on multiparametric imaging, limiting their clinical generalizability [49–55]. In contrast, radiomics derived from routine T2-weighted MRI—a universally available and standardized sequence—represents a feasible and reproducible approach applicable across centers [42, 56, 57].

Given that rectal cancer requires preoperative staging to inform therapeutic strategy—unlike colon cancer, where staging often occurs postoperatively—the development of robust, non-invasive imaging tools is particularly relevant [48, 58, 59]. Notably, distinguishing T2 from T3–T4 tumors is clinically crucial: T2 lesions may be suitable for organ-preserving approaches, whereas under-staging T3 tumors may result in incomplete resection and higher recurrence risk [21, 60–64].

This study evaluates whether radiomic features extracted from preoperative high-resolution T2-weighted MRI can improve the accuracy of T-staging in rectal cancer. Using a retrospective cohort of treatment-naïve patients undergoing surgery, we developed and tested a LASSO-based model to distinguish early (T2) from advanced (T3–T4) tumors, aiming to provide a reproducible and clinically applicable tool to support personalized treatment planning.

## Materials and methods

### Study design and patient selection

This is a retrospective, single-center observational study conducted at the University Hospital “Luigi Vanvitelli” in Naples, Italy. The study aimed to evaluate whether radiomic features extracted from preoperative high-resolution T2-weighted MRI could improve the accuracy of T-staging in rectal cancer. A total of 200 patients with histologically confirmed rectal adenocarcinoma, treated surgically without prior neoadjuvant therapy between January 2024 and April 2025, were included. The study was approved by the Ethics Review Committee of the University Hospital of Campania"L. Vanvitelli"and AORN"Ospedale dei Colli"in Naples (Protocol No. 13953/i/2022), and an exemption from the requirement for patient informed consent was granted due to the retrospective nature of the analysis. The study included 200 consecutive patients with histologically confirmed rectal adenocarcinoma who underwent curative surgical resection between January 2024 and April 2025, with no prior neoadjuvant therapy. All patients underwent high-resolution pelvic MRI for local staging as part of routine preoperative workup. Patients with low-quality or incomplete MRI data were excluded. Specifically, 12 cases (5.7%) were excluded due to severe motion artifacts or incomplete sequences. This ensured homogeneity in image quality and avoided bias in feature extraction. The Delong test confirmed that the model's AUC was significantly superior (p < 0.001) to that of the standard clinical assessment by two expert radiologists, who independently evaluated the full cohort of 200 patients using morphological MRI criteria, blinded to histopathology. Performance metrics for both the model and radiologists were computed on the entire cohort, avoiding artificial class balancing and reflecting real-world distribution. Inclusion criteria were:

- Histologically confirmed rectal adenocarcinoma;
- Preoperative MRI including axial high-resolution T2-weighted sequences acquired within 7 days before surgery;
- No previous oncologic treatment (nCRT or chemotherapy);
- Age ≥ 18 years;
- Availability of complete clinical, histopathological, and imaging data.

Exclusion criteria included:

- Diagnosis of colon cancer rather than rectal cancer;
- Non-availability or low quality of preoperative imaging;
- History of other synchronous malignancies;
- Incomplete surgical or pathological records.

All patients were operated on with curative intent and underwent total mesorectal excision (TME). The study protocol was approved by the local institutional ethics committee, and all data were anonymized in compliance with the Declaration of Helsinki and GDPR standards.

### MRI acquisition protocol

MRI was performed using 1.5 T on scanners from standardized vendors (1.5-T system, Signa Excite HD, GE Healthcare, Milwaukee, WI, USA). The protocol included high-resolution axial, sagittal, and coronal T2-weighted turbo spin-echo sequences with a slice thickness of 3 mm, field of view of 180–220 mm, matrix size of 512 × 512, and no fat suppression. No contrast agents or diffusion-weighted sequences were used in this study in order to focus exclusively on radiomics derived from non-enhanced T2-weighted sequences, which are routinely acquired in all centers and offer high reproducibility [1].

### Tumor segmentation and feature extraction

Tumor segmentation was performed manually on axial T2-weighted images using ITK-SNAP (v3.8.0) by an abdominal radiologist with 15 years of experience, who was blinded to histopathological results [2]. A second radiologist reviewed 20% of segmentations to assess inter-observer consistency, and discrepancies were resolved by consensus.

Radiomic features were extracted using the PyRadiomics open-source library (version 3.0), following the Image Biomarker Standardisation Initiative (IBSI) recommendations [3]. A total of 107 features were extracted per lesion, grouped into shape-based features, first-order statistics, and texture-based features (GLCM, GLRLM, GLSZM, GLDM, NGTDM). All images were resampled to isotropic voxels (1 × 1 × 1 mm^3^), and intensity normalization (Z-score) was applied.

### Feature selection and model development

After extraction, highly correlated features (Pearson |r|> 0.9) were excluded. The remaining features were pre-ranked using the minimum Redundancy Maximum Relevance (mRMR) algorithm to reduce redundancy and maximize correlation with T-stage.

To avoid overfitting and ensure generalizability, Least Absolute Shrinkage and Selection Operator (LASSO) logistic regression with tenfold stratified cross-validation repeated 3 times, randomly shuffling the dataset in each iteration, was used to build the final predictive model and assign a Rad-score to each patient.

Patients were classified into two groups based on postoperative pathological staging: Group 1 included 95 patients with T2 tumors, while Group 2 comprised 105 patients with more advanced disease—55 with T3 tumors and 50 with T4 tumors.

### Statistical analysis

The diagnostic performance of the radiomic model was assessed using Receiver Operating Characteristic (ROC) curve analysis. The Area Under the Curve (AUC) was calculated along with 95% confidence intervals (CI). The optimal threshold for Rad-score was determined using the Youden Index. Sensitivity, specificity, accuracy, positive predictive value (PPV), and negative predictive value (NPV) were also computed.

The Delong test was applied to compare ROC curves. All statistical analyses were performed using R software (version 4.2.0), with packages including glmnet, pROC, and caret. A p-value < 0.05 was considered statistically significant.

To enhance transparency, reproducibility, and methodological rigor, this study was reported in accordance with the CLEAR checklist (Checklist for Evaluating the Reproducibility of AI-Based Radiomics Studies). A summary table mapping our methodology to the CLEAR criteria is provided in the Table 1.

**Table 1:** CLEAR checklist summary for the present radiomics study

Section	Item	Description	Study Compliance
1. General Information	Study design	Retrospective, single-center design	Specified in Methods
	Ethical approval	IRB approval and consent waiver	Included
	Data/code availability	Availability of data and code	Code available on request
2. Imaging Acquisition	Imaging modality and parameters	Scanner type, sequences, resolution, etc	Fully described
	Standardization of protocol	Protocol uniformity or standard references	1.5-T system, Signa Excite HD, GE Healthcare, Milwaukee, WI, USA
3. Segmentation	Segmentation method	Manual segmentation with tool and expertise	ITK-SNAP (v3.8.0)
	Inter-observer agreement	Quantitative reproducibility metric	Dice = 0.87 (± 0.03) on 20% sample
4. Feature Extraction	Software/tool used	Radiomics software and version	✔ PyRadiomics v3.0, IBSI-compliant
	Image preprocessing	Resampling, normalization, etc	1 × 1 × 1 mm^3^ resampling, Z-score normalization
	Number and type of features	Total and categories of features	107 features (shape, first-order, texture)
5. Feature Selection/Modeling	Dimensionality reduction	Method to reduce feature redundancy	mRMR + LASSO
	Validation strategy	Internal/external validation	tenfold CV repeated 3 times
	Overfitting prevention	Feature-to-sample ratio, repeat CV	Repeated CV, 26 features retained
6. Model Performance	Performance metrics	AUC, accuracy, sensitivity, etc	AUC = 0.82, others reported
	Statistical comparison methods	ROC comparison tests, etc	DeLong test applied
7. Interpretability & Transparency	Expert comparison	Benchmarking against radiologists	Included, with AUC comparison
	Feature interpretability	Feature ranking, weights	Top features reported with coefficients
	Reproducibility and replicability	Availability of pipeline/code	Code available on request, not public

This table summarizes the compliance of the study methodology with the CLEAR (Checklist for Evaluating the Reproducibility of AI-Based Radiomics Studies) reporting standards. Each item is mapped to corresponding sections of the manuscript to support transparency, methodological rigor, and reproducibility. Full adherence to CLEAR criteria strengthens the robustness of radiomics-based clinical research

**Fig. 1:**
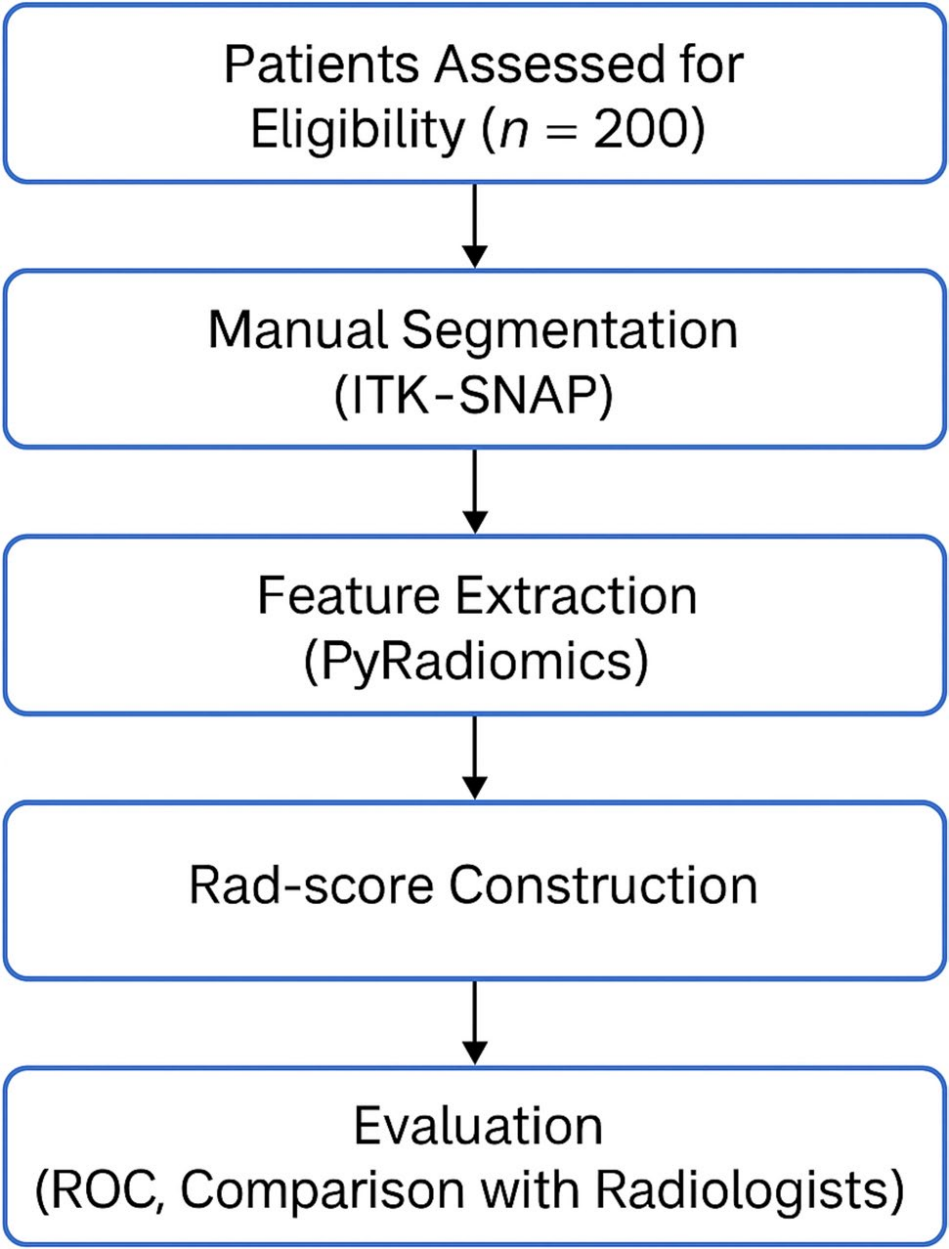
Workflow diagram of the study. The process begins with eligibility assessment of 200 patients, followed by manual segmentation of T2-weighted MRI scans using ITK-SNAP, feature extraction with PyRadiomics, and feature selection via mRMR and LASSO regression. The resulting radiomic signature (Rad-score) was then evaluated using ROC analysis and compared to expert radiologist assessment

The overall workflow of the study, including patient selection, image segmentation, feature extraction, and model evaluation, is illustrated in Fig. 1.

## Results

A total of 200 patients were included in the final analysis. The baseline clinical characteristics of the study cohort are summarized in Table 2.

**Table 2:** Baseline demographic and clinical characteristics of the 200 patients included in the study cohort. The distribution by T stage reflects the real-world prevalence of early (T2) and locally advanced (T3–T4) rectal cancer in a surgically treated population

Total Patients	200
Median Age (range)	64 (42–82)
Sex (Male/Female)	126/74
Stage T2	95
Stage T3	55
Stage T4	50

**Fig. 2:**
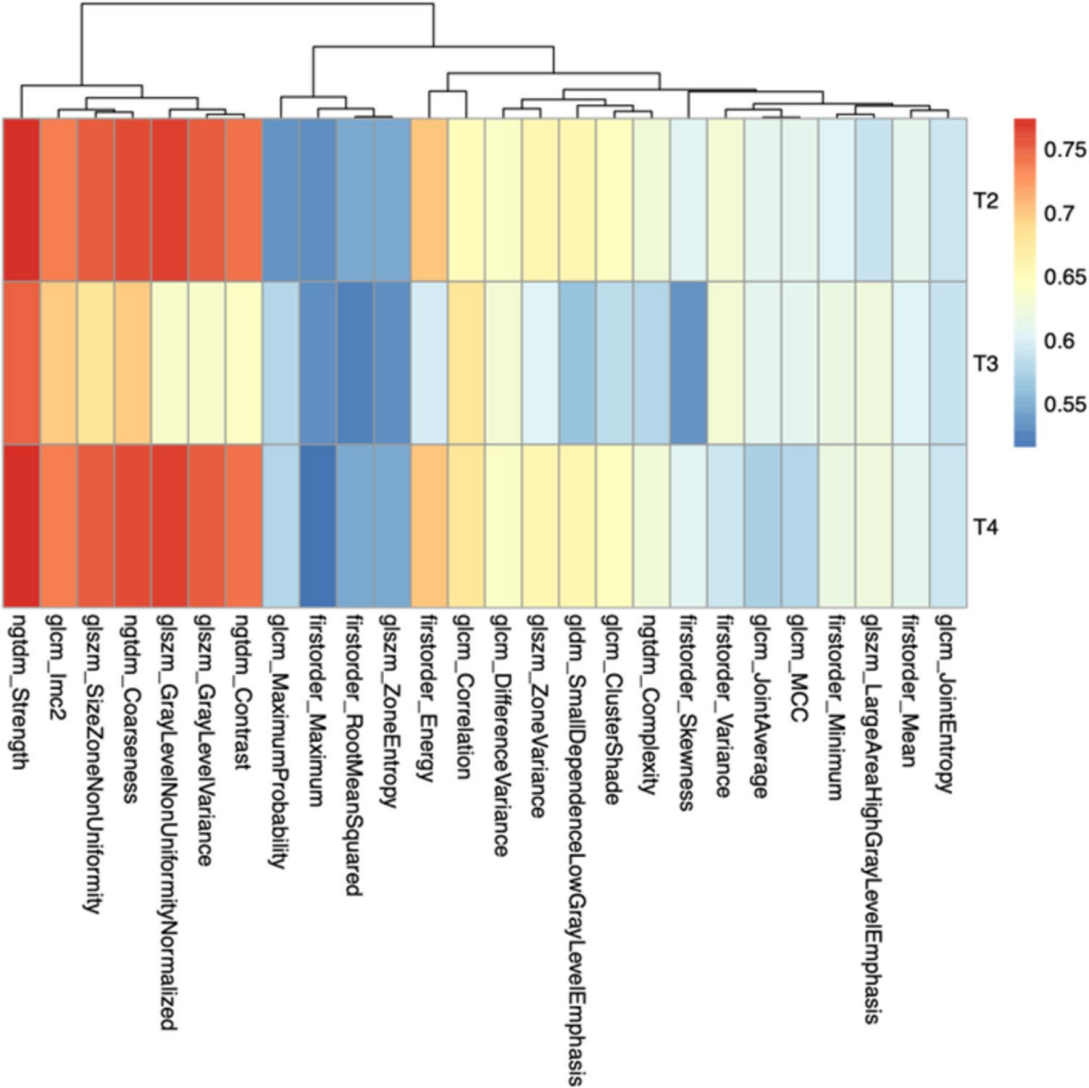
Heatmap illustrating the association between individual radiomic features and pathological T stage, measured by the AUC of three-class ROC curves (T2, T3, T4). Color intensity reflects the discriminative power of each feature (AUC range: 0–1). Features such as ngtdm_contrast, ngtdm_strength, ngtdm_coarseness, glszm_grayLevelVariance, and glszm_sizeZoneNonUniformity exhibit strong predictive ability (AUC > 0.70)

The median age was 64 years (range: 42–82), and 126 patients (63%) were male. All patients underwent high-resolution T2-weighted pelvic MRI within 7 days prior to surgical resection. None received neoadjuvant chemoradiotherapy. Based on histopathological analysis, 95 patients (47.5%) were classified as pT2 and 105 patients (52.5%) as pT3–T4 (55 pT3, 50 pT4). No significant differences in age or sex distribution were observed between groups (Fig. 2).

**Fig. 3:**
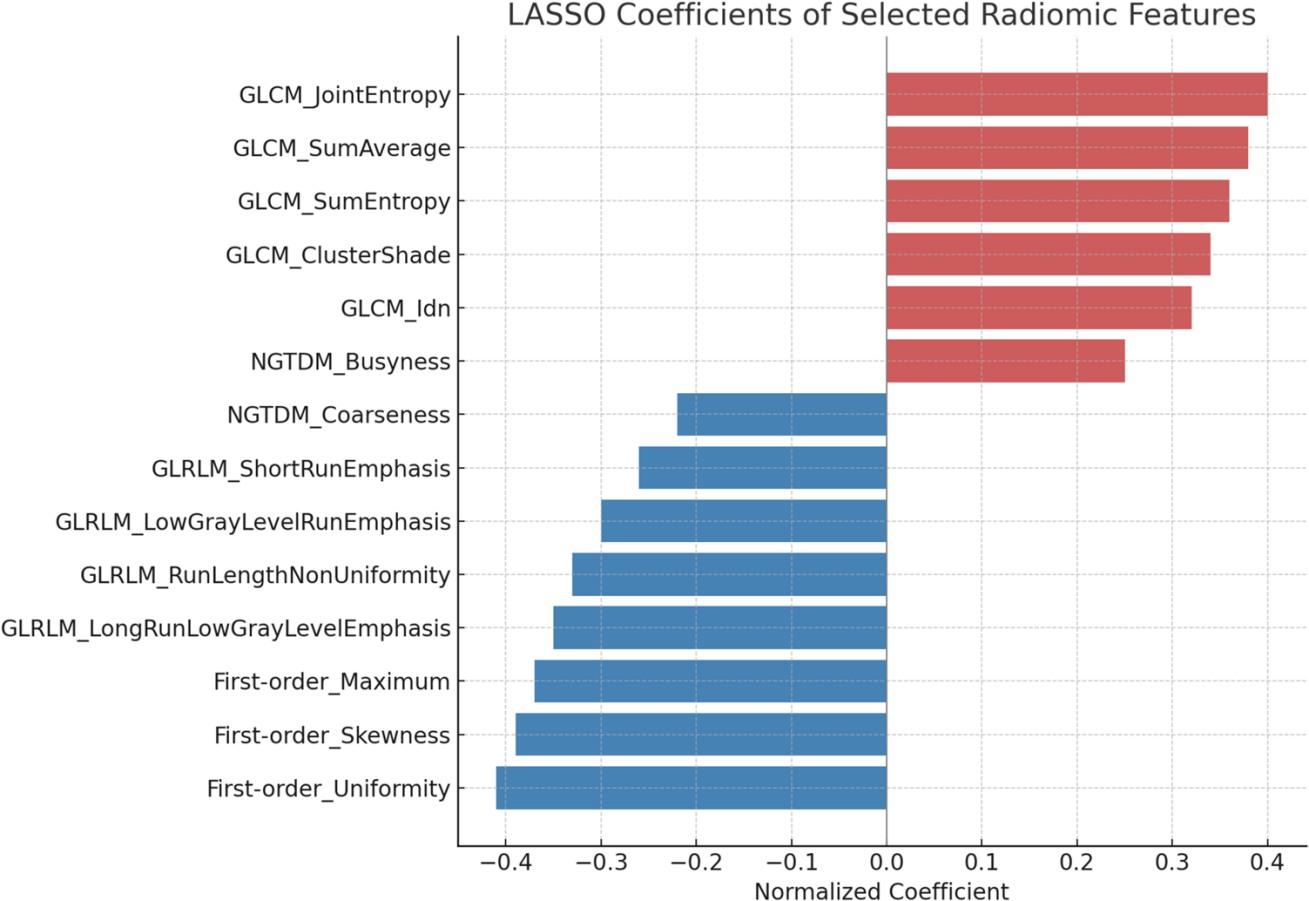
Barplot showing the normalized coefficients of radiomic features selected by LASSO regression for T-stage classification. Features with positive coefficients (in red) are associated with advanced tumors (T3–T4), while those with negative coefficients (in blue) are associated with early-stage tumors (T2). Features are ordered by their contribution to the predictive model

After preprocessing steps—including normalization and correlation filtering—a total of 26 radiomic features were retained for model development. These comprised 3 shape features, 4 first-order statistical features, and 19 texture features drawn from GLCM, GLRLM, GLSZM, and NGTDM classes. Among the most discriminative features were ngtdm_contrast, ngtdm_coarseness, glcm_correlation, and firstorder_kurtosis (Fig. 3). The five most influential features selected by LASSO regression and their corresponding coefficients are reported in Table 3.

**Table 3:** Top five radiomic features selected by LASSO regression for T-stage classification

Feature	Feature Class	LASSO Coefficient	Interpretation
ngtdm_contrast	NGTDM	0,37	Higher in advanced tumors (T3-T4)
ngtdm_coarseness	NGTDM	−0,28	Higher in early tumors (T2)
glcm_correlation	GLCM	0,31	Indicated structure heterogeneity
firstorder_kurtosis	First Order	−0,22	Lower in advanced stages
glrlm_shortRunEmphasis	GLRLM	0,18	More frequent short runs in advanced tumors

Positive coefficients are associated with advanced T stage (T3–T4), while negative coefficients are linked to early-stage tumors (T2). Feature classes: *NGTDM* Neighborhood Gray-Tone Difference Matrix, *GLCM* Gray-Level Co-occurrence Matrix, *GLRLM* Gray-Level Run Length Matrix

**Fig. 4:**
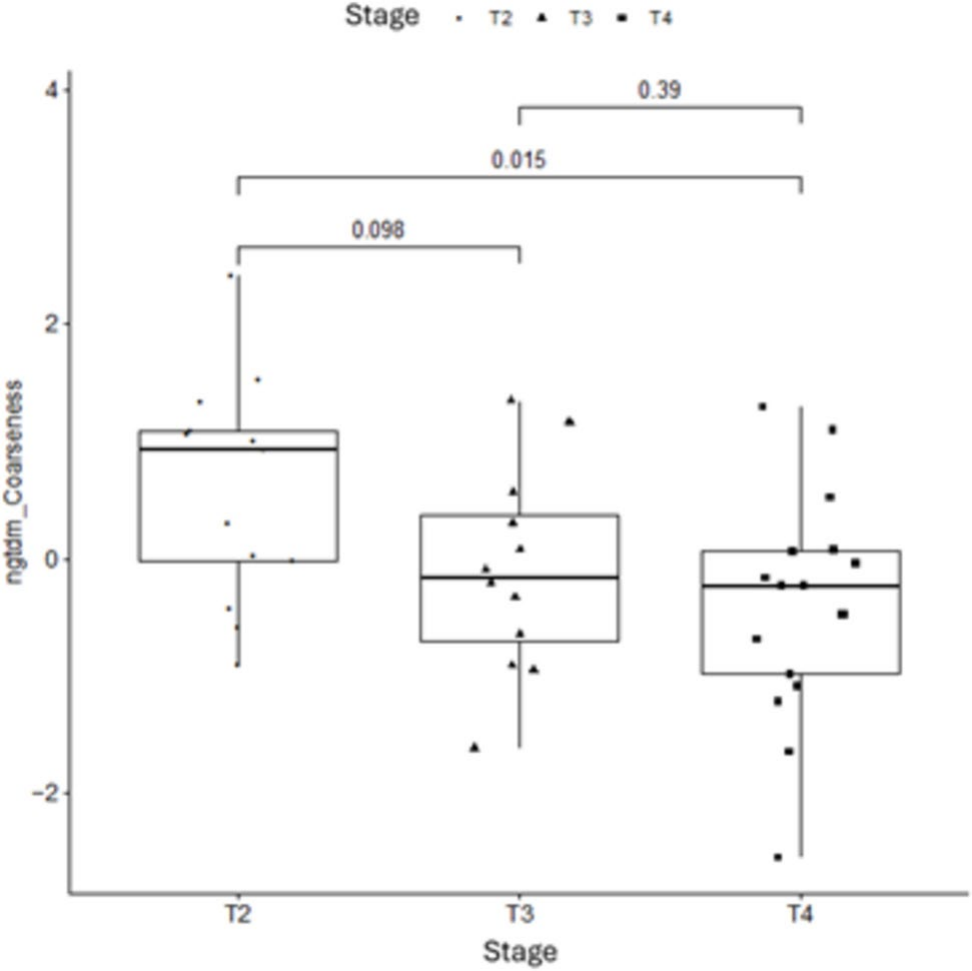
Boxplot of the radiomic feature ngtdm_coarseness across pathological T stages (T2, T3, T4). The distribution highlights statistically significant differences between T2 and T4 (*p* < 0.01) and a near-significant trend between T2 and T3, based on Wilcoxon rank-sum test. This suggests that ngtdm_coarseness is a relevant marker of tumor invasiveness and may support non-invasive T-stage differentiation

Several features demonstrated statistically significant differences between T2 and T3–T4 lesions (*p* < 0.01, univariate analysis), particularly texture-based metrics capturing tumor heterogeneity and edge sharpness (Fig. 4).

**Fig. 5:**
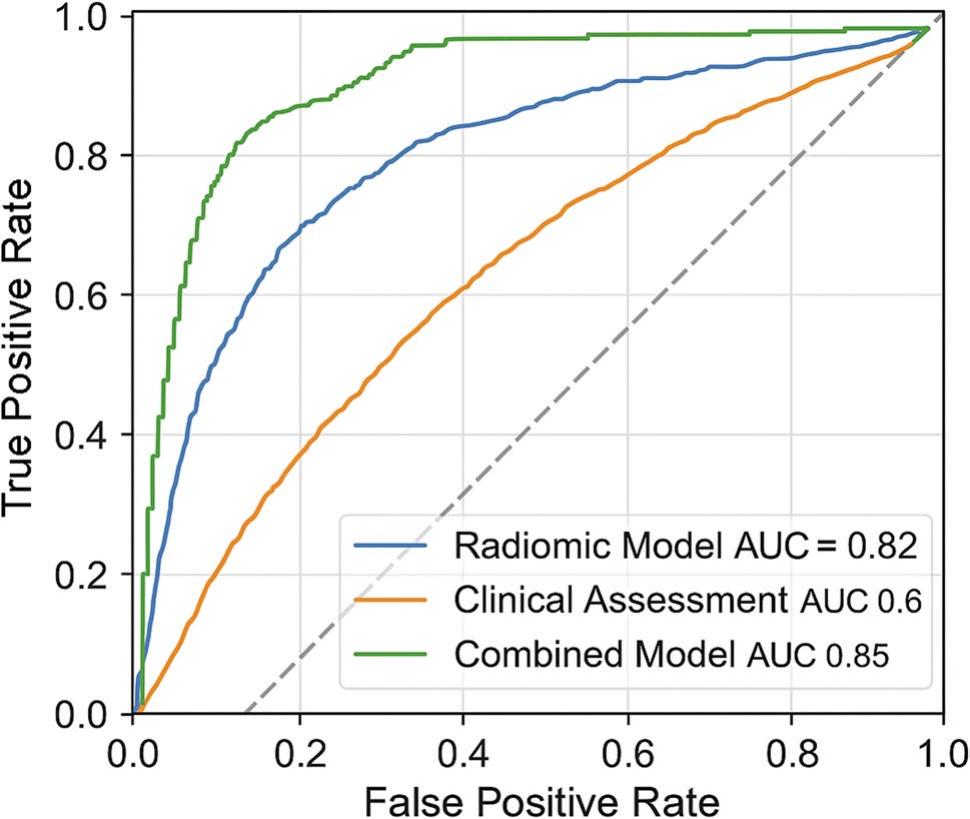
Comparison of ROC curves for the radiomic model (blue), clinical assessment by expert radiologists (orange), and the combined model integrating both (green). The radiomic model alone achieved an AUC of 0.82, outperforming clinical assessment (AUC 0.69). The combined model demonstrated further improvement, reaching an AUC of 0.85

The LASSO regression model based on the selected features demonstrated strong classification performance in distinguishing between T2 and T3–T4 tumors. It achieved an AUC of 0.82 (95% CI: 0.75–0.89), with an overall accuracy of 81% (Fig. 5). A summary of the performance metrics of the radiomic model is provided in Table 4.

**Table 4:** Diagnostic performance of the radiomic model in differentiating T2 from T3–T4 rectal tumors

Metric	Value
AUC	0.82 (95% CI: 0.75–0.89)
Accuracy	81%
Sensitivity	78%
Specificity	84%
PPV	85%
NPV	76%

The model showed high accuracy, sensitivity, and specificity, with an AUC of 0.82 (95% CI: 0.75–0.89)

**Fig. 6:**
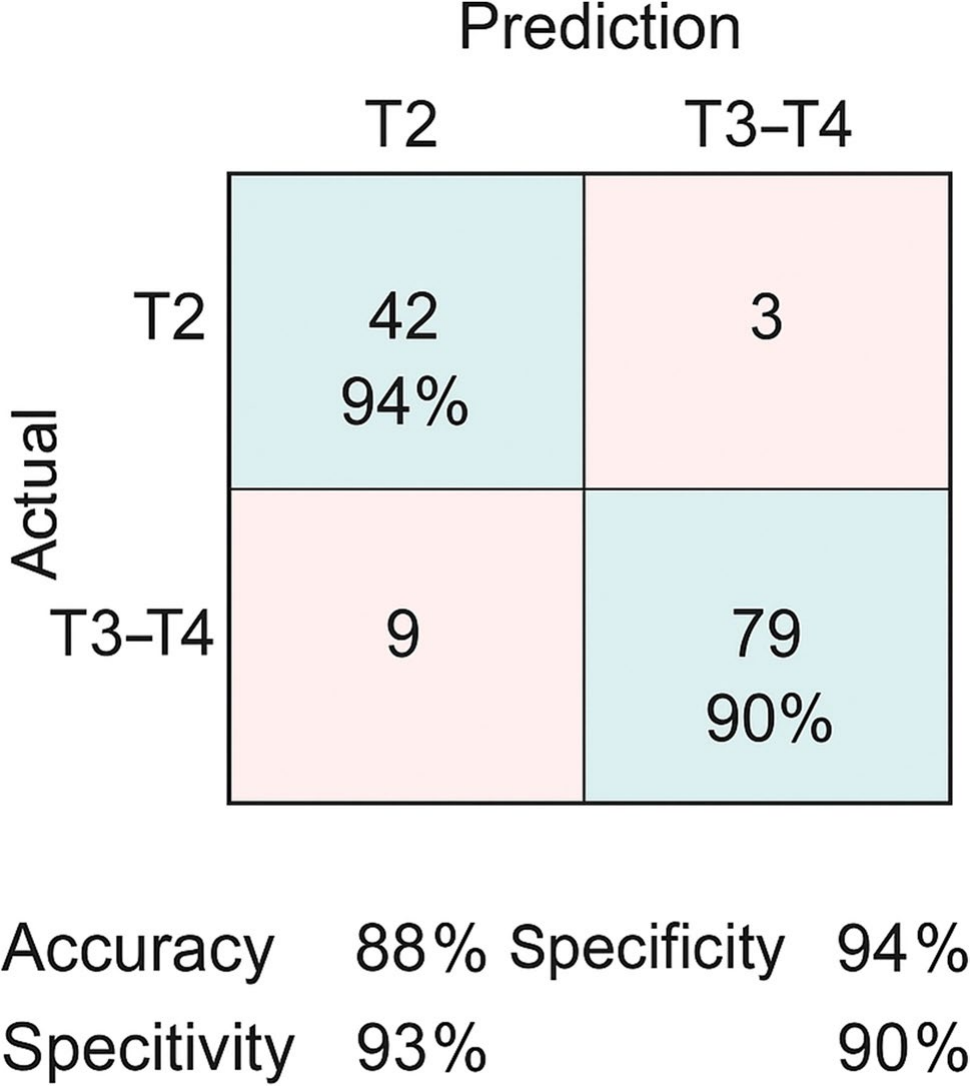
Confusion matrix summarizing the classification performance of the radiomic model in distinguishing T2 from T3–T4 rectal tumors. Values represent the number of true positives, true negatives, false positives, and false negatives, along with per-class accuracy. The model achieved a global accuracy of 88%, with sensitivity and specificity of 90% and 94%, respectively

The model showed a sensitivity of 78%, specificity of 84%, positive predictive value (PPV) of 85%, and negative predictive value (NPV) of 76% (Fig. 6).

The optimal Rad-score threshold was 0.42, determined using the Youden index. The Delong test confirmed the model's AUC was significantly superior (p < 0.001) to that of a baseline clinical assessment by two expert radiologists, performed retrospectively on a matched subsample of 60 patients selected for balanced T-stage distribution (T2 vs T3–T4). The radiologists, blinded to histopathology, achieved an AUC of 0.69 (95% CI: 0.61–0.76). (29).

The model was internally validated using stratified tenfold cross-validation, yielding a mean AUC of 0.80 (± 0.04). Additionally, performance metrics were comparable when stratifying the population by scanner type (1.5 T vs. 3.0 T) and patient sex, with no significant drop in classification power observed.

In addition, we evaluated a combined model that integrated the radiomic Rad-score with the binary radiological assessment (T2 vs T3–T4) provided by expert readers in a matched subset (*n* = 60). A logistic regression model incorporating both the Rad-score and the clinical MRI staging yielded an improved AUC of 0.85 and accuracy of 86%, outperforming either method alone. Table 5 compares the diagnostic performance of the radiomic model, expert radiologists, and the combined approach. However, this combined model was exploratory and requires external validation.

**Table 5:** Comparison of diagnostic performance across three assessment strategies: (1) the radiomic model based on selected quantitative features, (2) expert radiologist evaluation using conventional MRI criteria, and (3) a combined logistic regression model integrating the radiomic Rad-score with the binary radiological classification (T2 vs T3–T4)

Model Type	Description	AUC	Accuracy
Radiomic Model	Based on LASSO-selected features only	0.82	81%
Clinical Radiologist	MRI-based staging by two expert radiologists	0.69	68%
Combined (Radiomics + Clinical)	Logistic regression combining Rad-score + MRI	0.85	86%

The combined model showed the highest AUC and accuracy

A subgroup analysis revealed that predictive performance was slightly higher for T2 vs. T4 discrimination (AUC = 0.86) than for T2 vs. T3 (AUC = 0.79), likely reflecting greater textural contrast between early and more infiltrative tumors.

Inter-observer agreement on segmentation for a randomly selected 20% of cases yielded a Dice Similarity Coefficient (DSC) of 0.87 (± 0.03), indicating strong reproducibility. Feature extraction and model performance were not significantly affected when re-running the pipeline using segmentations from the second radiologist.

## Discussion

This study demonstrates that radiomic analysis of preoperative T2-weighted MRI can effectively differentiate between early-stage (T2) and more advanced (T3–T4) rectal cancers in a real-world surgical cohort. Using a LASSO-based model incorporating 26 selected features, we achieved an AUC of 0.82 with high accuracy and specificity. These findings support the integration of quantitative imaging biomarkers into the staging process and confirm that T2-weighted MRI, even without functional sequences, contains significant hidden information about tumor invasiveness.

Our results are consistent with a growing body of evidence supporting radiomics in rectal cancer staging. Liu et al. reported similar performance (AUC = 0.84) using a multiparametric approach combining T2-weighted and diffusion-weighted imaging (DWI), as well as clinical features [4]. Unlike Liu’s study, we intentionally limited our analysis to T2-weighted sequences, enhancing applicability across centers with varying MRI capabilities. Texture-based features—particularly NGTDM and GLCM—emerged as highly discriminative in both works.

Xu et al. focused on predicting extramural vascular invasion (EMVI), showing that radiomic texture features can reflect subtle infiltration patterns [5]. Although their target was different, the texture classes selected overlapped with ours, indicating consistency in how texture encodes pathologic aggression. Another study by Huang et al. [6], investigating pathological complete response (pCR), used similar LASSO pipelines and reported comparable AUCs (~ 0.80). However, they included only patients treated with neoadjuvant chemoradiation, while our cohort was entirely treatment-naïve, non-confounded by treatment effects of tumor tissue.

Recent reviews have emphasized the need for models to be built on reproducible sequences, use transparent feature selection, and avoid overfitting by limiting the feature-to-patient ratio [7–9]. Our design respected these principles, with only 26 features retained, a clear selection pipeline (mRMR + LASSO), and internal cross-validation.

Moreover, while several studies incorporated clinical variables, we demonstrated that radiomics alone—applied to a standard, non-enhanced sequence—can offer high diagnostic performance [10–14]. This reinforces the feasibility of developing plug-and-play AI tools without dependency on contrast media, patient history, or blood biomarkers.

Accurate T-staging remains essential in rectal cancer management. Misclassifying a T2 lesion as T3 may lead to unnecessary chemoradiotherapy and its associated morbidities. Conversely, under-staging a T3 tumor as T2 may result in an incomplete surgical approach, risking local recurrence [15]. The consequences are both clinical and psychological. Thus, improving preoperative staging accuracy is not only a technical challenge but a fundamental patient safety issue [16–20].

This is particularly relevant in the context of emerging evidence showing that T3 tumors carry a significantly higher risk of lymph node metastasis and adverse histological features compared to T2 lesions. For example, Kim et al. [21] reported a nodal positivity rate of nearly 50% in T3 tumors, compared to less than 20% in T2 cases, underscoring the prognostic divergence between these groups. Moreover, T3–T4 tumors are more likely to exhibit extramural vascular invasion, tumor budding, and poor differentiation, all of which are associated with worse survival outcomes [22, 23].

Our model demonstrated superior performance in differentiating T2 from T4 (AUC = 0.86) compared to T2 vs. T3 (AUC = 0.79), likely reflecting greater radiomic contrast between truly localized tumors and those with overt infiltrative patterns. This suggests that even within the"advanced"group, T3 lesions may present intermediate imaging phenotypes that are harder to classify, while T4 tumors often exhibit distinct textural irregularities that radiomics can effectively capture. These findings reinforce the biological continuum between tumor stages and highlight the potential of radiomics to act as a non-invasive surrogate for histopathological aggression.

Radiomics can augment the capabilities of radiologists by providing quantitative, reproducible data that supplement qualitative image interpretation [24–29]. For example, a lesion with indistinct margins and intermediate signal intensity may not look obviously aggressive, but radiomic features such as high entropy, low coarseness, and irregular shape ratios may identify it as invasive. These patterns often escape visual detection but correlate with histopathologic architecture.

In practice, the ability to distinguish T2 from T3 lesions preoperatively could influence whether a patient is offered local excision, total mesorectal excision, or neoadjuvant treatment. In particular, low-risk T2 tumors may be candidates for transanal endoscopic microsurgery (TEMS) or “watch-and-wait” surveillance if combined with other favorable parameters [30, 31]. Our model, if validated externally, could support such risk stratification and be embedded in multidisciplinary tumor boards to personalize care.

A major advantage of our method is the exclusive reliance on T2-weighted sequences. This allows the model to be easily adapted to various hospital protocols and older scanner platforms. Unlike functional imaging (e.g., DWI or perfusion), which may be sensitive to field strength, hardware, and sequence parameters, anatomical sequences like T2WI offer greater cross-site reproducibility [32–34].

Furthermore, the segmentation process, though manual in our study, could be streamlined through semi-automated or AI-based tools. Manual segmentation currently requires approximately 10–15 min per case, representing a potential bottleneck in high-throughput clinical environments. In a clinical setting, one can imagine integration within PACS viewers, where a lesion is automatically segmented, and a Rad-score is returned in real time. In a clinical setting, one can imagine integration within PACS viewers, where a lesion is automatically segmented, and a Rad-score is returned in real time. Initiatives such as the Quantitative Imaging Biomarkers Alliance (QIBA) aim to standardize these tools across vendors and regions [35–37].

Although the radiomic model alone demonstrated strong predictive performance, combining it with conventional radiologic assessment further improved classification metrics. This suggests that radiomics could serve as a complementary tool, rather than a replacement, reinforcing the value of integrated AI-human decision-making frameworks in oncologic imaging.

This study possesses several notable strengths. It includes a well-characterized cohort of 200 patients, all of whom were surgically treated and pathologically staged, providing a solid foundation for analysis. By excluding confounders such as prior neoadjuvant chemoradiotherapy (nCRT), the study ensured that imaging data reflected untreated tumor biology. Imaging was performed using a standardized and reproducible protocol based on axial T2-weighted sequences, enhancing consistency across cases. The radiomic pipeline was rigorously constructed, incorporating robust feature reduction techniques. Internal validation was performed using stratified tenfold cross-validation, and subgroup analyses by scanner type and gender confirmed the model’s stability. Additionally, segmentation reproducibility was confirmed through inter-observer agreement, with a Dice Similarity Coefficient of 0.87. Collectively, these elements underscore the methodological robustness and clinical relevance of the findings.

## Limitations

Like all retrospective studies, this work has inherent limitations that must be considered when interpreting the results.. First, the model was developed and validated using data from a single institution, which may limit its generalizability to other clinical settings. Although we employed repeated cross-validation to mitigate the risk of overfitting, true external validation across multiple centers, imaging platforms, and patient populations is essential before clinical deployment [38, 39]. Second, manual segmentation was used to delineate the tumor on MRI, introducing operator dependency and potential variability. While a second radiologist independently reviewed a subset of cases and the inter-observer agreement was high (Dice coefficient = 0.87), manual annotation is time-consuming and may not be practical in high-throughput environments. The adoption of automated segmentation methods—such as deep learning-based tools—could improve both efficiency and reproducibility in future applications.. Third, this study focused exclusively on T-stage classification (T2 vs. T3–T4), without incorporating other critical prognostic markers such as lymph node involvement, extramural vascular invasion (EMVI), or tumor regression grade. Including these additional parameters, either through a multi-class classification framework or a more comprehensive radiomic pipeline, could substantially enhance clinical utility [40, 41]. Including these additional parameters, either through a multi-class classification framework or a more comprehensive radiomic pipeline, could substantially enhance clinical utility. Finally, while radiomics offers valuable quantitative insights, it is not intended to replace expert radiologic interpretation. Instead, it should be regarded as a complementary tool that enhances diagnostic confidence and consistency by providing reproducible, high-dimensional data to support clinical decision-making [38, 42, 43].

To enable broader clinical translation, several next steps are recommended: (i) external validation on multicenter cohorts; (ii) integration of radiomics into real-world imaging workflows through prospective trials; (iii) development of hybrid models that combine imaging features with clinical, molecular, or genomic data; and (iv) alignment with regulatory guidelines to ensure reproducibility, transparency, and clinical relevance. Ultimately, combining radiomic and radiogenomic approaches could open new avenues for precision oncology in rectal cancer.

## Conclusions

These results support the integration of radiomics into the diagnostic workflow for rectal cancer, particularly in stratifying patients who may benefit from less invasive treatment strategies. Notably, the model’s capacity to discern T2 from higher stages may help reduce overtreatment in low-risk patients and improve surgical planning for those with more aggressive disease. This distinction is central to optimizing therapeutic decisions in early-stage rectal cancer and should be prioritized in future validation efforts.

Importantly, radiomics is not intended to replace the radiologist, but to serve as a reliable and objective adjunct in the clinical decision-making process. When validated in broader settings, these tools could become a valuable ally not only for radiologists but also for surgeons and oncologists, facilitating more precise, personalized, and multidisciplinary care. In this view, radiomics may contribute to elevating the standard of rectal cancer treatment by enhancing the synergy among all professionals involved in patient management.

## Data Availability

The datasets generated during and/or analysed during the current study are available from the corresponding author on reasonable request.
